# Telephone-Based Smoking Cessation Counseling Service: Satisfaction and Outcomes in Vietnamese Smokers

**DOI:** 10.3390/healthcare11010135

**Published:** 2022-12-31

**Authors:** Quy-Chau Ngo, Lan Phuong Thi Doan, Giap Van Vu, Thu-Phuong Phan, Hanh Thi Chu, Anh Tu Duong, Quan-Hoang Vuong, Manh-Tung Ho, Minh-Hoang Nguyen, Thu-Trang Vuong, Tham Thi Nguyen, Hien Thu Nguyen, Anh Hai Tran Nguyen, Cyrus S. H. Ho, Roger C. M. Ho

**Affiliations:** 1 Department of Internal Medicine, Hanoi Medical University, Hanoi 100000, Vietnam; 2 Respiratory Center, Bach Mai Hospital, Hanoi 100000, Vietnam; 3 Centre for Interdisciplinary Social Research, Phenikaa University, Yen Nghia, Ha Dong, Hanoi 100803, Vietnam; 4 Ritsumeikan Asia Pacific University, Beppu City 874-8577, Oita Prefecture, Japan; 5 Sciences Po Paris, Campus de Dijon, 21000 Dijon, France; 6 Institute for Global Health Innovations, Duy Tan University, Da Nang 550000, Vietnam; 7 Faculty of Nursing, Duy Tan University, Da Nang 550000, Vietnam; 8 Institute of Health Economics and Technology (iHEAT), Hanoi 100000, Vietnam; 9 Department of Psychological Medicine, Yong Loo Lin School of Medicine, National University of Singapore, Singapore 119228, Singapore; 10 Institute for Health Innovation and Technology (iHealthtech), National University of Singapore, Singapore 119077, Singapore

**Keywords:** smoking, QUITLINE, smoking relapse, smoking quit, satisfaction, telephone-based

## Abstract

Background: As a method to acknowledge the devastating health and economic impacts of tobacco usage worldwide, telephone-based tobacco cessation counseling services have emerged as a potential tool to aid people in their quitting process. This study explores the satisfaction of smokers who use the QUITLINE service and factors associated with their quit attempts and cessation. Methods: A cross-sectional survey of 110 participants was conducted from June to July 2016 at the Respiratory Center at Bach Mai Hospital, Hanoi, Vietnam. Multivariate logistic regression was used, and it was found that the percentage of people quitting smoking increased after using the service. Results: In total, 65.5% of participants were completely satisfied with the counseling service. The mean score of staff/s capacity/responsiveness, motivation, and service convenience were 4.37 ± 0.78, 4.30 ± 0.81, and 4.27 ± 0.66, respectively. The smoking relapse rate was relatively high at 58.3%, which mainly resulted from cravings and busy work (26.2% and 14.3%, respectively). A higher satisfaction score in “Staffs’ capacity and responsiveness” was negatively associated with “ever tried to quit smoking in consecutive 24 h” and actually quit smoking after receiving counseling. Meanwhile, a higher score in the “Motivation” domain was positively associated with both quit attempt indicators as well as actually quitting smoking after receiving counseling (OR = 9.48; 95%CI = 2.27; 39.57). Conclusions: These results suggest that it is crucial for decision makers to place more focus on countermeasures for smoking relapse and to strengthen the capacity of staff, especially in motivating clients. Interventions should also be maintained throughout a long period of time to prevent relapse.

## 1. Introduction

Despite efforts to alleviate the burden of tobacco use worldwide, smoking remains the leading cause of death. According to the World Health Organization’s report on trends in the prevalence of tobacco, tobacco smoking is the most common cause of non-communicable diseases (NCDs) [1], accountable for over 6 million deaths per year [1], and is the determinant of health complications such as cardiovascular disease, deteriorated respiratory system, or lung cancer [2,3]. In addition to negative health effects, financial losses due to tobacco abuse are also evident, such as an increase in healthcare costs or loss in productivity and performance quality [4,5,6]. On average, the total economic cost of smoking accounts for 5.7% of global health expenditure, equivalent to USD 1.9 trillion in 2012 [7]. This problem is even more severe for developing countries, whose population growth will account for 97% of the predicted worldwide rate in 2030 [8], which will, in turn, increase the number of smokers [8]. A study by Mathers and Loncar projected that the number of mortalities attributable to smoking would decrease by 9% in high-income countries and double from 3.4 to 6.8 million by 2030 in lower-income countries [9].

Fortunately, smoking cessation has been found to reverse much damage in both health quality and personal finance [10]. Death rate corresponding with prolonged smoking can be reduced by up to 90% if the practice is adopted before the age of 40 [11]. Smoking cessation is beneficial not only for the smoker but also for surrounding people as it decreases the chance of secondhand smoke exposure, especially for children and pregnant women [12,13]. Even though smoking cessation measures such as cigarette taxation, restriction of marketing and promotion related to tobacco products, prohibition of public smoking, and mandated placement of warning labels on tobacco products [14] are becoming increasingly popular worldwide, the effectiveness of such interventions varies between settings, especially in developing countries where the applicability and affordability of frameworks are often limited [15]. Studies on determinants of smoking cessation around the world receive complex results as researchers have investigated a variety of factors, including socio-demographic, socioeconomic, cultural, psychological, physical, alcohol consumption status, etc. [16,17,18,19,20,21]. In Vietnam, while there are ongoing efforts to identify predictors of smoking cessation, a small body of research was conducted on real-life data which was able to reflect the true scope of smoking problems. According to the Global Adult Tobacco Survey (GATS) in 2015, tobacco smoking prevalence in Vietnam was 22.5%, with 45.3% among males and 1.1% among the female population [22]. The rate of people who reported exposure to secondhand smoke was relatively high at 59.9% [4]. The total economic cost due to tobacco was estimated to be approximately USD 1,173 million in 2011, equivalent to nearly 1% of Vietnam’s Gross Domestic Product (GDP) [23]. 

In 2005, the World Health Organization (WHO) held a Framework Convention on Tobacco Control (FCTC) on the prevention and reduction of tobacco use, and the MPOWER package, which selects reduction measures, was released in 2008 [24]. Although the number of smokers in Vietnam seemed to decline from 2010 to 2015, the change was not significant enough to infer a downward trend. There was almost no change in the cessation rate (29.3% in 2010 and 29.0% in 2015), and the proportion of smokers making a quit attempt in the past 12 months dropped significantly from 55.3% to 39.6% [22,25]. The halt in progress regarding national smoking cessation and smoking quit attempts suggest that more effective cessation programs should be developed while existing interventions should also be strengthened [22]. Age, the number of cigarettes smoked per day, and previous quit attempts can be used as predictive variables of quitting confidence and planning to quit [26]. In 2015, the national counseling service QUITLINE proposed toll-free telephone-based therapy provided by professional counselors as an intervention method available for people who want to quit smoking and avoid relapse. Information on the national QUITLINE (national anti-smoking education and communication programs) can be accessed via social media, the most popular platform utilized by youths, and registration for counseling can also be facilitated by telephone. The telephone-based counseling model for smoking cessation has been proposed and implemented in other developed countries, such as the United States and the United Kingdom [27,28]. It was found to substantially increase the long-term quitting outcome of adult smokers who called willingly and correlated with sustainable abstinence after multiple sessions [29]. The implementation of telephone counseling could be facilitated alongside other interventions as a complimentary measure and could be initiated from both sides, the counselor or the help-seeking smoker [30]. Therefore, among numerous tobacco interventions, free telephone counseling with a skilled counselor to discuss quit plans is often regarded as the most common, cost-effective approach to improve self-efficacy and motivation to achieve long-term abstinence [24,31]. Despite the effectiveness of the telephone-based model on smoking cessation outcomes, there is a lack of literature on the association between satisfaction with smoking cessation services and smoking quit attempts/cessation. According to the study which was conducted in Vietnam, 88.5% of participants were satisfied with the quality of the provided services, 74.3% felt more confident about quitting, 81.7% took early action via their first quit attempt, and 18.3% reported a more than 7-day abstinence period at the time of the survey [31]. Therefore, this article aims to expand this research topic by exploring the effects of customer satisfaction on the cessation and quit attempts of smokers using telephone-based counseling services in Vietnam.

## 2. Methods 

### 2.1. Study Design and Participants

A cross-sectional study was conducted at the Respiratory Center at Bach Mai Hospital from June 1 to July 31, 2016. To support tobacco users to quit tobacco use, the Respiratory Center at Bach Mai Hospital established the QUITLINE service. The service, offered by 10 trained drug treatment counselors, was free and available to patients every day of the week from 8:00 a.m. to 10:00 p.m.

In terms sampling, we used the convenience sampling technique. The inclusion criteria were as follows: (1) participants aged 18 years old or above, (2) consulted by QUITLINE staff, (3) used QUITLINE services previously (but currently do not use), and (4) available to take part in a phone interview. Patients invited to participate in the study were fully informed of the content, purpose, and benefits of participating in the study. The response rate was 100.0%.

### 2.2. Data Collection and Measurement

Before receiving counseling sessions, participants were requested to complete a standardized questionnaire survey.

#### 2.2.1. Demographic and Tobacco Use Characteristics

Information regarding individual characteristics of participants (age on set, sex, education, and marital status), tobacco use before using the QUITLINE service (18006606, hosted by the Respiratory Center at Bach Mai Hospital, Hanoi, Vietnam) (age at the first smoking, the lowest/highest number of cigarettes per day, and whether living with other smokers in the family or not), and history of smoking cessation (number of quit attempts and quitting methods) were collected via closed-ended questions.

Additionally, we asked participants to report smoking patterns and quit attempts after using previous QUITLINE services, including whether they were current smokers or not, had ever tried to quit smoking after counseling sessions, reasons for relapse, number of cigarettes per day, number of quit attempts, whether they had ever tried to quit smoking in consecutive 24 h, and improvement in health conditions after using previous QUITLINE services.

#### 2.2.2. Patient Satisfaction Measures

We used a systematic process to develop a patient satisfaction scale. First, we investigated national and international literature to identify potential patient satisfaction items with smoking cessation counseling services. We determined that the instrument should comprise three domains: (1) convenience of service, (2) capacity of counseling staff, and (3) capacity to motivate quitting attempts. Then, we performed a focus group discussion with patients seeking smoking cessation support, medical staff, and researchers to investigate the instrument’s face validity. In the next step, through focus group discussions, a list of potential issues was produced and selected depending on the relevance of each item. To examine its appropriateness through culture, language, and administration, we piloted the service with 20 patients. Each item was marked from 1 “Complete dissatisfaction” to 5 “Complete satisfaction”. Each domain’s score was derived by averaging the scores of domain-related items. Hence, the total score of each domain ranged from 1 to 5. Participants who provided a higher score indicated a higher level of satisfaction. 

### 2.3. Statistical Analysis

STATA software version 12.0 was used to analyze the data (Stata Corp. LP, College Station, TX, USA). Exploratory factor analysis (EFA) was used to investigate the construct validity of the satisfaction measurement. Associated factors were extracted by principal component analysis. A threshold was established at an eigenvalue of 0.5 when the curve flattened out. The screen determined the threshold. To reorganize items on the scale, we used Orthogonal Varimax rotation with Kaisers’ normalization to raise the interpretability of these factors. A cut-off point of 0.5 was used to calculate factor loadings. We also conducted cross-loading on one item before assigning it to the proper domain based on the nature of the query as well as the overarching dimension. The internal consistency reliability of measurement was assessed through Cronbach’s alpha. 

We used multivariate logistic regression to identify the associated factors with ever tried to quit smoking after counseling (Yes/No), ever tried to quit smoking consecutively for 24 h (Yes/No), and actual quitting smoking after receiving counseling (Yes/No). To generate the final models, stepwise backward techniques were integrated with these regression models, which employed a *p*-Value of log-likelihood of 0.2 as the criterion for selecting variables. The statistical significance level was set at *p* < 0.05.

## 3. Results

Among 110 patients, most were male (99.1%) with a mean age of 44.9 (SD = 13.0) years old. The majority of patients had a high school education or above (78.2%) and had a spouse/partner (87.3%) (Table 1).

All of the patients were smokers before participating in this study. The mean age at first smoking was 18.5 (SD = 3.9) years old. The previous lowest and highest number of cigarettes used per day were 10.4 (SD = 9.1) and 23.9 (SD = 10.8), respectively. One-third of the participants had never attempted to quit smoking before (34.6%). Among those attempting to quit, self-help was the most common method (86.1%) (Table 2).

Regarding current smoking patterns, 49.1% reported that they were smokers. In total, 76.4% and 81.8% tried to quit smoking after the counseling session and quit smoking in the following 24 h, respectively. The mean number of quit attempts was 1.6 (SD = 1.0) times. Reasons for relapse included craving (26.2%), friends (8.3%), bad mood (9.5%), and busy work (14.3%) (Table 3).

Table 4 demonstrates that three domains were detected after conducting EFA for the patient satisfaction scale concerning the counseling service, namely “Staffs’ capacity and responsiveness”, “Motivation”, and “Service convenience”, with Cronbach’s alpha values of 0.9468, 0.9094, and 0.9019, respectively. The highest number of respondents who were completely satisfied was 67.3% in “Staff hears respectfully and supports to solve my problems”, while the lowest percentage was 34.6%% in “Location of counseling service is convenient”. Participants provided the lowest score regarding “Service convenience” (Mean = 4.27, SD = 0.66) and the highest score regarding “Staffs’ capacity and responsiveness” (Mean = 4.37; SD = 0.78).

Table 5 shows the associated factors with quit attempts and current smoking status. A higher number of quit attempts before the counseling session was associated with trying to quit smoking after counseling compared to never (OR = 5.71; 95%CI = 1.59; 20.49). Those of higher age, not living with smokers in the family, were less likely to try quitting smoking in the following 24 h, while those of higher age at first smoking were more likely to try this behavior. The upper age range was also observed to be associated with a current smoker (OR = 3.13; 95%CI = 1.12; 8.76).

Regarding satisfaction, higher satisfaction scores in “Staffs’ capacity and responsiveness” were negatively associated with “ever tried to quit smoking in consecutive 24 h” and actually quitting smoking after receiving counseling. Meanwhile, a higher score in the “Motivation” domain was positively associated with both quit attempt indicators and actually quitting smoking after receiving counseling (OR = 9.48; 95%CI = 2.27; 39.57). 

## 4. Discussion

This study was one of the first to explore the impacts of clients’ satisfaction with smoking cessation in Vietnam. Based on data collected from users of the QUITLINE service at the Respiratory Center in Bach Mai hospital, this study found that even though the prevalence of smoking quit attempts and cessation increased after counseling, there was still a considerably high relapse rate reported. The satisfaction of motivation provided by the service was positively associated with attempting to quit smoking after counseling during the first 24 h and actual smoking cessation, while service convenience did not influence smoking quit attempts and cessation. 

Similar to other studies indicating the benefits and effectiveness of smoking cessation services, particularly the QUITLINE service [32,33,34], our study found that the percentage of people attempting to quit smoking increased from 65.4% to 76.4% after using the service. Not only did the attempts to quit tobacco increase, but the cessation rate after consultation (50.9%) was also higher than the cessation rate reported in the GATS (29%) [22]. Nevertheless, it was notable that the smoking relapse rate reported by our respondents was still relatively high at 58.3% and was brought about by craving and busy work (26.2% and 14.3%, respectively). Multiple but scattered periods of smoking abstinence might be the reason for the high prevalence of relapse, as the highest risk period for relapse was found to be within the first year after smoking cessation [35,36]. Thus, it was implied that the QUITLINE service should be developed as a sustainability-oriented service, meaning regular and constant follow-ups should be provided to prevent clients from smoking relapse. 

Compared to other studies on client satisfaction with QUITLINE [37,38], besides conventional variables including the convenience of the service, staff’s capacity, and responsiveness, our study introduced a new measurement of client satisfaction, namely the capacity of staff to motivate quitting smoking. Client satisfaction with the staff’s ability to motivate was found to positively correspond with the rate of quitting attempts after counseling during the first 24 h and success in quitting smoking. Though not through staff encouragement, smoker motivation to quit was found to be a crucial determinant of favorable quitting outcomes in previous studies [39,40]. In our study, it is reasonable to infer that higher perceived client satisfaction corresponds with greater motivation received from counseling. Thus, this result underlines the importance of strengthening staff capacity to motivate clients.

Among the three aspects of client satisfaction with the QUITLINE service, the convenience of service was found to be the least correlated, while the capacity and responsiveness of staff were the most influential factors. This pattern is similar to that in the Republic of Korea, where factors related to the convenience of service, such as counseling time assigned per call, were the variables least associated with satisfaction [38]. Indeed, as the telephone platform is considered the optimal platform with high access across different demographics, there is little room for improvement in convenience or accessibility. Instead, efforts to enhance the quality of QUITLINE services should be directed at staff training or content quality. High satisfaction associated with staff capacity and responsiveness can be explained by the fact that better service delivery often results in better understanding, practice, and trust in the advised intervention [41]. Therefore, clinicians in tobacco cessation counseling facilities are required to have information, education, and communication (IEC) materials as well as a computer system to effectively manage clients’ information and deliver consultations [24,42,43]. Despite this, the capacity of staff to motivate clients was found to be significantly reduced after an unsuccessful cessation. Not receiving enough support might be the reason for higher rates of relapse at almost 60%. Three primary reasons underlying relapse among smokers were identified as stress, craving, and environment [39]. As the staff in QUITLINE services were often only paraprofessional counselors, they tended not to have sufficient knowledge and expertise for in-depth support or complications [44]. Therefore, the staff of QUITLINE services should receive training not only in care delivery and quality but also in expanding their expertise in tobacco cessation strategies and response to complications. 

Among the variables investigated concerning quit attempts and smoking cessation, age, the number of quit attempts before counseling, the status of the person’s health after consultation, age of first smoke, and living with smokers were found to be predictors of smoking quit attempts and cessation outcomes. Our results showed that age was negatively associated with the effort to quit smoking in the first 24 h and smoking cessation, which is contrary to another study in Poland [45] and consistent with a study in India [46] and a study by Ömer Alkan et al. [47]. Particularly, the findings of these studies indicated that as age increased, the possibility of relapse to tobacco use after cessation decreased. Most people who failed to cease smoking were in the 18–44 age group, and those who were successful in quitting were in the group aged 65 and above [48]. Therefore, our study raised concern regarding the negative consequences of early smoking by highlighting that people who started tobacco consumption at an early age were less likely to be motivated toward quitting tobacco in the first 24 h and suggested that prevention campaigns and awareness-raising movements should be implemented at a young age rather than focusing on mitigation later in life.

It is reasonable that the number of quit attempts before counseling positively correlated with those after counseling, as already having the commitment to stop smoking facilitated by advice from counselors might be a strong influence on clients’ quit attempts after service [24,39,49]. This finding is consistent with a previous study in Turkey [50]. Particularly, the findings of this study showed that the probability of people intending to quit tobacco use who have tried to quit before was more than that of those who have not. Furthermore, the study by Omer Alkan [50] indicated that awareness of anti-smoking messages has an effect on the intention to quit tobacco use. It was suggested that the probability of intention to quit tobacco use of people being aware of anti-smoking messages was more than that of those who were not. The success rate was also higher for those who quit tobacco use with help. Similar to previous results, our results point out that people who were trying to quit smoking after counseling were more likely to perceive better health conditions, which in turn motivated them to quit smoking during the first 24 h.

There are certain limitations to this study. First, the collection of the smoking status of the participants was self-reported and might be subject to recall bias. Second, the questionnaire was adjusted from the original English scale to fit with Vietnam’s culture, language, and administrative characteristics of the study site [51]. Thirdly, the sample size was relatively small, with a total of 110 respondents, most of whom were males. Therefore, the results of this study could not be generalized and should be referred to as a guideline. Finally, on a methodological note, as scientists around the world have pointed out the problem with the classical method of statistical inference, this study can benefit from the Bayesian approach [52,53].

## 5. Conclusions

Our study highlighted the importance of customer satisfaction with QUITLINE services in promoting and supporting smoking quit attempts and smoking cessation. More specifically, smokers motivated by QUITLINE’s staff were more likely to engage in tobacco cessation after counseling, especially for the first 24 h, or to quit smoking completely. It is also notable that those who attempted to quit smoking after counseling experienced better health conditions and thus were more motivated to quit smoking in the during the first 24 h. In light of these determinants, policies should encourage the use of telephone-based QUITLINE services and approach tobacco cessation from a client satisfaction viewpoint, namely by improving staff capacity, responsiveness, and ensuring constant follow-ups on smoking quit attempts.

## Figures and Tables

**Table 1:** Demographic characteristics of respondents (n = 110).

Characteristics	Frequency (n)	Percentage (%)
**Gender (Male)**		
Male	109	99.1
Female	1	0.9
**Age groups (years old)**		
18–30	16	14.6
31–40	24	21.8
41–50	37	33.6
51–60	18	16.4
>60	15	13.6
**Age, Mean (SD)**	44.9 (13.0)	
**Education**		
<High school	24	21.8
High school	44	40.0
>High school	42	38.2
**Marital status**		
Single	14	12.7
Having a spouse/partner	96	87.3

**Table 2:** Tobacco use and quit attempts before receiving counseling services.

Characteristics	Frequency (n)	Percentage (%)
**Number of quit attempts**		
Never	38	34.6
1–3 times	49	44.6
4–6 times	19	17.3
More than 6 times	4	3.6
**Quitting methods**		
Self-help	62	86.1
Direct counseling from health staff	3	4.2
Nicotine replacement therapy	4	5.6
Others	3	4.2
**Living with other smokers in the family**		
Yes	63	57.3
No	47	42.7
**Age at first smoking, Mean (SD)**	18.5 (3.9)	
**The lowest number of cigarettes per day, Mean (SD)**	10.4 (9.1)	
**The highest number of cigarettes per day, Mean (SD)**	23.9 (10.8)	

**Table 3:** Smoking patterns after receiving counseling service.

Characteristics	Frequency (n)	Percentage (%)
**Ever tried to quit smoking after the counseling session**		
Yes	84	76.4
No	26	23.6
**Ever tried to quit smoking in 24 h**		
Yes	90	81.8
No	20	18.2
**Actual quit smoking**		
Yes	54	49.1
No	56	50.9
**Reasons for relapse**		
Craving	22	26.2
Friends invited	7	8.3
Mood	8	9.5
Work	12	14.3
**Improvement of health condition**		
Much better	10	9.1
Better	41	37.3
Not change	51	46.4
Worse	8	7.3
**Number of cigarettes per day, Mean (SD) (n = 54)**	16.1 (9.5)	
**Number of quit attempts, Mean (SD) (n = 86)**	1.6 (1.0)	

**Table 4:** Factor loadings of satisfaction measures.

Items	% Completely Satisfied	Factor Loadings of Measure Domains		
		Staff’s Capacity and Responsiveness	Motivation	Service Convenience
**Location of counseling service is convenient**	34.6			0.7851
Daytime for counseling is appropriate	39.1			0.8288
Weekday for counseling is appropriate	40.0			0.839
Counseling in smoking cessation provides hope to quit	44.6		0.7084	
Confident in introducing the counseling service to other people	54.6		0.8405	
Staff were trained well in counseling	57.3	0.5058		
Staff explain the necessary information clearly	59.1	0.6383		
Staff act respectfully and provide support to solve my problems	67.3	0.7549		
Waiting time between booking and receiving service is appropriate	63.6			0.7773
Received necessary information for smoking cessation	60.9	0.6162		
Felt comfortable when answering staff’s questions	62.7	0.7297		
Received support motivated me to quit smoking	59.1		0.6399	
Length of each counseling session is sufficient	38.2	0.7177		
% floor		0.0	0.9	0.0
% ceiling		30.0	38.2	29.1
Reliability (Cronbach’s alpha)		0.9468	0.9094	0.9019
Domains scores (Mean ± SD)		4.37 ± 0.78	4.30 ± 0.81	4.27 ± 0.66
General satisfaction				
% Completely satisfied with the counseling service	65.5			

**Table 5:** Associated factors with quit attempts and current smoking status.

Characteristics	Ever Tried to Quit Smoking after Counseling (Yes/No)		Ever Tried to Quit Smoking in the Following 24 h (Yes/No)		Actually Quit Smoking after Receiving Counselling (Yes/No)	
	OR	95%CI	OR	95%CI	OR	95%CI
**Age group**						
18–30 years old			1		1	
31–40 years old			0.20 *	0.04; 1.10	0.32 **	0.11; 0.89
>60 years old			0.11 **	0.01; 0.86		
**Marital status**						
Having a spouse/ partner					1	
Single					0.37	0.10; 1.32
**Number of quit attempts before the counseling session**						
Never	1		1			
1–3 times	5.71 ***	1.59; 20.49	2.71	0.66; 11.08		
>3 times	2.59	0.67; 9.95				
**Improvement of health condition**						
Much better	1		1			
Not change	0.32 *	0.10; 1.06	0.03 ***	0.00; 0.26		
**Age at the first smoking**			1.33 **	1.05; 1.69		
**Living with other smokers in the family**						
Yes	1		1			
No	0.38	0.12; 1.23	0.10 **	0.02; 0.64		
**Staff’s capacity and responsiveness**	0.24	0.03; 1.81	0.02 **	0.00; 0.47	0.18 **	0.04; 0.74
**Motivation**	10.89 **	1.53; 77.45	54.06 ***	2.75; 1,063.47	9.48 ***	2.27; 39.57
**Service convenience**	0.29	0.04; 1.91				

*** *p* < 0.01, ** *p* < 0.05, * *p* < 0.1; OR: Odds ratio; CI: Confidence Interval.

## Data Availability

The datasets used and/or analyzed during the current study are available from the corresponding author upon reasonable request.

## References

[1] WHO (2015). WHO Global Report on Trends in Prevalence of Tobacco Smoking. https://apps.who.int/iris/bitstream/handle/10665/156262/9789241564922_eng.pdf.

[2] Proctor R.N. (2001). Tobacco and the global lung cancer epidemic. Nat. Rev. Cancer.

[3] Roy A., Rawal I., Jabbour S., Prabhakaran D. (2017). Tobacco and cardiovascular disease: A summary of evidence. Cardiovascular, Respiratory, and Related Disorders.

[4] Max W. (2001). The financial impact of smoking on health-related costs: A review of the literature. Am. J. Health Promot..

[5] Halpern M.T. (2001). Impact of smoking status on workplace absenteeism and productivity. Tob. Control.

[6] Collins D.J., Lapsley H.M. (2008). The Costs of Tobacco, Alcohol and Illicit Drug Abuse to Australian Society in 2004/05.

[7] Goodchild M., Nargis N., Tursan d’Espaignet E. (2018). Global economic cost of smoking-attributable diseases. Tob. Control.

[8] Consultancy.uk (2015). 97% of Population Growth to Be in Developing World. https://www.consultancy.uk/news/2191/97-percent-of-population-growth-to-be-in-developing-world.

[9] Mathers C.D., Loncar D. (2006). Projections of global mortality and burden of disease from 2002 to 2030. PLoS Med..

[10] Pirie K., Peto R., Reeves G.K., Green J., Beral V., Collaborators M.W.S. (2013). The 21st century hazards of smoking and benefits of stopping: A prospective study of one million women in the UK. Lancet.

[11] Jha P., Ramasundarahettige C., Landsman V., Rostron B., Thun M., Anderson R.N., McAfee T., Peto R. (2013). 21st-century hazards of smoking and benefits of cessation in the United States. N. Engl. J. Med..

[12] WHO (2019). Fact Sheet about Health Benefits of Smoking Cessation. https://www.who.int/tobacco/quitting/benefits/en/.

[13] Rosen L.J., Noach M.B., Winickoff J.P., Hovell M.F. (2012). Parental smoking cessation to protect young children: A systematic review and meta-analysis. Pediatrics.

[14] Forouzanfar M.H., Afshin A., Alexander L.T., Anderson H.R., Bhutta Z.A., Biryukov S., Brauer M., Burnett R., Cercy K., Charlson F.J. (2016). Global, regional, and national comparative risk assessment of 79 behavioural, environmental and occupational, and metabolic risks or clusters of risks, 1990–2015: A systematic analysis for the Global Burden of Disease Study 2015. Lancet.

[15] Abdullah A.S.M. (2004). Promotion of smoking cessation in developing countries: A framework for urgent public health interventions. Thorax.

[16] Yang J.J., Song M., Yoon H.-S., Lee H.-W., Lee Y., Lee S.-A., Choi J.-Y., Lee J.-k., Kang D. (2015). What are the major determinants in the success of smoking cessation: Results from the health examinees study. PLoS ONE.

[17] Castro Y. (2016). Determinants of smoking and cessation among Latinos: Challenges and implications for research. Soc. Personal. Psychol. Compass.

[18] Holm M., Schiöler L., Andersson E., Forsberg B., Gislason T., Janson C., Jogi R., Schlünssen V., Svanes C., Torén K. (2017). Predictors of smoking cessation: A longitudinal study in a large cohort of smokers. Respir. Med..

[19] Poghosyan H., Moen E.L., Kim D., Manjourides J., Cooley M.E. (2019). Social and structural determinants of smoking status and quit attempts among adults living in 12 US states, 2015. Am. J. Health Promot..

[20] Ussher M., Kakar G., Hajek P., West R. (2016). Dependence and motivation to stop smoking as predictors of success of a quit attempt among smokers seeking help to quit. Addict. Behav..

[21] Lertsinudom S., Pranboon S., Chantapasa K., Hansuri N. (2018). Motivation and satisfaction in smoking cessation service. Res. Soc. Adm. Pharm..

[22] Ministry of Health (2015). Global Adult Tobacco Survey (GATS): Fact Sheet Vietnam 2015.

[23] Pham T.H.A., Thu L.T., Ross H., Quynh Anh N., Linh B.N., Minh N.T. (2014). Direct and indirect costs of smoking in Vietnam. Tob. Control.

[24] Hoang V.M., Tran T.N., Vu Q.M., Nguyen T.T.M., Le H.C., Vu D.K., Tran T.A., Nguyen B.N., Vu V.G., Nguyen M.C. (2016). Tobacco control policies in Vietnam: Review on MPOWER implementation progress and challenges. Asian Pac. J. Cancer Prev..

[25] Ministry of Health of Vietnam (2010). Global Adult Tobacco Survey (GATS): Fact Sheet Vietnam 2010.

[26] Ngo C., Chiu R., Chu H., Vu G., Nguyen Q., Nguyen L., Tran T., Nguyen C., Tran B., Latkin C. (2018). Correlated factors with quitting attempts among male smokers in Vietnam: A QUITLINE-based survey. Int. J. Environ. Res. Public Health.

[27] Matkin W., Ordóñez-Mena J.M., Hartmann-Boyce J. (2019). Telephone counselling for smoking cessation. Cochrane Database Syst. Rev..

[28] Zhu S.-H., Anderson C.M., Johnson C.E., Tedeschi G., Roeseler A. (2000). A centralised telephone service for tobacco cessation: The California experience. Tob. Control.

[29] Lichtenstein E., Glasgow R., Lando H., Ossip-Klein D., Boles S. (1996). Telephone counseling for smoking cessation: Rationales and meta-analytic review of evidence. Health Educ. Res..

[30] Boyle R.G., Solberg L.I., Asche S.E., Maciosek M.V., Boucher J.L., Pronk N.P. (2007). Proactive recruitment of health plan smokers into telephone counseling. Nicotine Tob. Res..

[31] Ngo C.Q., Phan P.T., Vu G.V., Pham Q.T.L., Chu H.T., Pham K.T.H., Tran B.X., Do H.P., Nguyen C.T., Tran T.T. (2019). Impact of a Smoking Cessation Quitline in Vietnam: Evidence Base and Future Directions. Int. J. Environ. Res. Public Health.

[32] Borland R., Segan C. (2006). The potential of quitlines to increase smoking cessation. Drug Alcohol Rev..

[33] Schauer G.L., Bush T., Cerutti B., Mahoney L., Thompson J.R., Zbikowski S.M. (2013). Use and effectiveness of Quitlines for smokers with diabetes: Cessation and weight outcomes, Washington state Tobacco Quit Line, 2008. Prev. Chronic Dis..

[34] Vickerman K.A., Zhang L., Malarcher A., Mowery P., Nash C. (2015). Cessation outcomes among quitline callers in three states during a national tobacco education campaign. Prev. Chronic Dis..

[35] Hughes J.R., Peters E.N., Naud S. (2008). Relapse to smoking after 1 year of abstinence: A meta-analysis. Addict. Behav..

[36] García-Rodríguez O., Secades-Villa R., Flórez-Salamanca L., Okuda M., Liu S.-M., Blanco C. (2013). Probability and predictors of relapse to smoking: Results of the National Epidemiologic Survey on Alcohol and Related Conditions (NESARC). Drug Alcohol Depend..

[37] Grunseit A.C., Gwizd M., Lyons C., Anderson C., O’Hara B.J. (2018). Polite, professional, practical: What drives caller ‘satisfaction’ with the New South Wales Quitline, Australia: Satisfaction and quitline callers. Drug Alcohol Rev..

[38] Jeong B.Y., Lim M.K., Yun E.H., Oh J.K., Park E.Y., Shin S.H., Park E.C. (2012). User Satisfaction as a Tool for Assessment and Improvement of Quitline in the Republic of Korea. Nicotine Tob. Res..

[39] Buczkowski K., Marcinowicz L., Czachowski S., Piszczek E. (2014). Motivations toward smoking cessation, reasons for relapse, and modes of quitting: Results from a qualitative study among former and current smokers. Patient Prefer. Adherence.

[40] McCaul K.D., Hockemeyer J.R., Johnson R.J., Zetocha K., Quinlan K., Glasgow R.E. (2006). Motivation to quit using cigarettes: A review. Addict. Behav..

[41] Armin J.S., Nair U., Giacobbi P., Povis G., Barraza Y., Gordon J.S. (2020). Developing a guided imagery telephone-based tobacco cessation program for a randomized controlled trial. Tob. Use Insights.

[42] Government of V. (2013). Decree 176/2013/ND-CP.

[43] Shelley D., Nguyen L., Pham H., VanDevanter N., Nguyen N. (2014). Barriers and facilitators to expanding the role of community health workers to include smoking cessation services in Vietnam: A qualitative analysis. BMC Health Serv Res.

[44] Lichtenstein E., Zhu S.-H., Tedeschi G.J. (2010). Smoking cessation quitlines: An underrecognized intervention success story. Am. Psychol..

[45] Kaleta D., Korytkowski P., Makowiec-Dąbrowska T., Usidame B., Bąk-Romaniszyn L., Fronczak A. (2012). Predictors of long-term smoking cessation: Results from the global adult tobacco survey in Poland (2009–2010). BMC Public Health.

[46] Srivastava S., Malhotra S., Harries A.D., Lal P., Arora M. (2013). Correlates of tobacco quit attempts and cessation in the adult population of India: Secondary analysis of the Global Adult Tobacco Survey, 2009–2010. BMC Public Health.

[47] Alkan Ö., Demir A. (2019). Investigation and detection of risk factors related to the period without tobacco consumption. Addicta Turk. J. Addict..

[48] Kim Y.-J. (2014). Predictors for successful smoking cessation in Korean adults. Asian Nurs. Res..

[49] West R. (2017). Tobacco smoking: Health impact, prevalence, correlates and interventions. Psychol. Health.

[50] Alkan O., Demir A. (2020). Factors Affecting the Intention to Quit among Women Smokers in Turkey. Asian Pac. J. Cancer Prev..

[51] Vuong Q.-H., Bui Q.-K., La V.-P., Vuong T.-T., Nguyen V.-H.T., Ho M.-T., Nguyen H.-K.T., Ho M.-T. (2018). Cultural additivity: Behavioural insights from the interaction of Confucianism, Buddhism and Taoism in folktales. Palgrave Commun..

[52] Amrhein V., Greenland S., McShane B. (2019). Scientists rise up against statistical significance. Nature.

[53] La V.P., Vuong Q.H. bayesvl: Visually Learning the Graphical Structure of Bayesian Networks and Performing MCMC with ‘Stan’ (Version 0.8.5). The Comprehensive R Archive Network (CRAN) 2019. https://cran.r-project.org/web/packages/bayesvl/index.html.

